# Predictive Value of Plasma Copeptin Level in Children with Acute Heart Failure

**DOI:** 10.1007/s00246-022-02909-w

**Published:** 2022-05-09

**Authors:** Doaa El Amrousy, Dina Abdelhai, Mohammed Nassar

**Affiliations:** 1 Pediatric Department, Faculty of Medicine, Tanta University, Tanta, Egypt; 2 Clinical Pathology Department, Faculty of Medicine, Tanta University, Tanta, Egypt

**Keywords:** Copeptin, Heart failure, Mortality, Pediatric, Predictive value

## Abstract

We investigated the ability of copeptin level to predict adverse outcome in pediatric heart failure (HF) and correlated copeptin level with various clinical and echocardiographic data. This cohort study was carried out on forty children with clinical picture of acute HF as the patient group and forty healthy children of matched age and sex as the control group. Echocardiographic examination and plasma copeptin level were performed for all included children at admission. Patients were followed up for 6 months for mortality or readmission. Plasma copeptin level was significantly higher in the patient group (16.2 ± 5) pmol/L compared to the control group (4.1 ± 2.3) pmol/L, *P* ˂0.001. Moreover, copeptin level was positively correlated with Ross classification, being the highest in patients with class IV (19.6 ± 3.9) pmol/L compared to those with class III (15.2 ± 4) pmol/L and class II (10.4 ± 1.5) pmol/L. Copeptin levels were significantly higher in patients with bad prognosis (21.2 ± 4.1) pmol/L compared to those with good prognosis (14.5 ± 4.1) pmol/L, *P* ˂0.001. Copeptin level had a significant positive correlation with age, heart rate, respiratory rate, and ROSS classification. On the contrary, copeptin level had a significant negative correlation with left ventricular fraction shortening and diastolic function. Copeptin at cut-off value of ≥ 19.5 pmol/L yielded a sensitivity of 75% and a specificity of 93% to predict adverse outcome in children with HF. Plasma copeptin level has a good prognostic value to predict adverse outcome in pediatric heart failure. Moreover, copeptin correlate well with the severity of pediatric HF.

## Introduction

Heart failure (HF) is one of the most common causes of morbidity and mortality in pediatrics worldwide [1]. Independent of the etiology, neurohormonal activation plays an important role in the pathophysiology of the development and progression of HF [2]. The renin angiotensin aldosterone system (RAAS) as well as vasopressin (VP) and the sympathetic nervous system are activated in cases of HF leading to salt and water retention, vasoconstriction, and increased heart rate [3].

Low cardiac output as well as increased osmolality in HF lead to stimulation of the release of VP hormone from the posterior pituitary which has both antidiuretic and vasoconstrictor properties and thus regulating water balance and hemodynamics of the body [4]. Early prediction of high risk patients with HF who need more intense strategy of management is crucial to improve the survival rate. Several biomarkers have been studied to predict the severity of HF, adverse outcome, and survival in children with HF [5, 6].

Copeptin is synthesized with VP and released in equimolar amounts. It is considered as a surrogate biomarker for VP due to its stability and longer half-life than VP [7]. Plasma copeptin is found to correlate well to VP levels in plasma [8]. Increased copeptin level has been reported in adults with HF [9, 10]. Furthermore, copeptin was reported to have a good prognostic value to predict mortality and adverse outcome in adults with HF in several studies [11, 12]. However, the prognostic value of copeptin in pediatric HF has not been evaluated yet.

The aim of this study was to evaluate the ability of copeptin level to predict adverse outcome in pediatric HF and to correlate copeptin level with various clinical and echocardiographic data.

## Patients and Methods

This cohort study was carried out at Pediatric Cardiology unit, Tanta University hospital during the period from January 2019 to December 2020. Forty children with clinical picture of acute HF were enrolled as the patient group. Forty healthy children of matched age and sex served as the control group. Patients were classified according to modified Ross classification of HF in infants and children to class I, II, III, and IV [13]. The study was approved by the local ethical committee of faculty of medicine, Tanta University. Written informed consents were obtained from parents of all children involved in the study.

**Inclusion Criteria**: Children aged less than 18 years with manifestations of acute HF either due to acquired or congenital heart disease (CHD).
**Exclusion Criteria**: children with neuromuscular mitochondrial disease, ischemic heart disease, cancer, diabetes mellitus, diabetes insipidus, obesity, central nervous system diseases, hepatic, or renal disease.

HF was diagnosed both clinically by symptoms and signs of HF as tachycardia, tachypnea, enlarged tender liver, dyspnea, orthopnea, edema, sweating, and feeding intolerance, and radiologically by chest X ray and echocardiography.

All participants were subjected to full history taking and thorough clinical examination including weight, heart rate (HR), respiratory rate (RR), and complete local cardiac examination. Routine laboratory evaluation including complete blood count, liver function tests, and kidney function tests was performed.

Conventional Echocardiographic examination was performed using a Vivid seven ultrasound machine (GE Medical System, Horten, Norway, with a 3.5 MHz multifrequency transducer) to evaluate left ventricular (LV) systolic function using two-dimensional and M mode at parasternal long axis view. LV systolic function was evaluated as: LV fractional shortening (FS) = LV end-diastolic dimension (LVEDD) − LV end-systolic dimension (LVESD) / (LVEDD) × 100%. Left ventricular diastolic function was measured using pulsed Doppler through the mitral valve in the form of mitral E/A ratio where E wave is a peak early filling velocity and A wave is a peak late filling velocity.

Plasma levels of copeptin was measured using a sandwich enzyme-linked immunosorbent assay test (ELISA). Two milliliters of venous blood sample was collected from each patient in an EDTA vacutainer tube labelled with the patient name. The blood was mixed gently and centrifuged for separation of plasma, which was stored at − 20 °C till the time of analysis. Plasma Copeptin levels were analyzed with an ELISA kit (Shanghai SunRed Bio-Tech. CO. LTD, China). The intra-and inter-assay coefficients of variation (CV) were ˂ 10 and ˂ 12% respectively.

Echocardiographic examination and plasma copeptin levels were measured at the time of admission. Patients were followed up for 6 months for adverse outcomes such as mortality and re-admission to the hospital. Good prognosis was defined as no mortality, readmission, or complications during the period of follow-up, while poor prognosis was defined as the incident of death, readmission, or complications during the period of follow-up. Complications were defined as the occurrence of HF or chest infection.

## Statistical analysis

Statistical analysis was performed using SPSS V.20. For quantitative data, the mean and standard deviation (SD) were calculated. For qualitative data, number and percentages were calculated. Comparison of qualitative data between two groups was performed using Chi-square test (*χ*^2^). Comparison of the means between the two groups was performed using Student *t*-test. Comparison of the mean between more than two groups was performed using one way analysis of variance (ANOVA) test. Correlation between variables was evaluated using Pearson’s correlation coefficient (*r*). The Receiver Operating Characteristic (ROC) curve was drawn to detect the prognostic value of copeptin to predict adverse outcome among children with HF at different cutoff points. *P* < 0.05 is considered significant.

## Results

The study included 40 children with HF as the patient group with mean age 3.8 ± 2.7 years, they were 15 male and 25 female. Forty healthy children of matched age and sex served as the control group with mean age 4 ± 2.9 years, they were 17 male and 23 female. There was no significant difference between the patient and control group as regards age and sex. Weight, LV FS, and mitral E/A ratio were significantly lower in the patient group compared to the control group. HR and RR were significantly higher in the patient group compared to the control group. Thirteen out of the 40 patient (32.5%) had HF due to cardiomyopathy, while 27 out of 40 patients (67.5%) had HF due to CHD. Patients with CHD (27 patients) were diagnosed as; eight patients with ventricular septal defect, five patients with patent ductus arteriosus, seven patients with complete atrioventricular canal, four patients with transposition of great arteries, two patients with coarctation of aorta, one patient with pulmonary stenosis. Plasma copeptin level was significantly higher in the patient group (16.2 ± 5) compared to the control group (4.1 ± 2.3), *P* ˂0.001. (Table 1).

**Table 1:** Demographic, clinical, and laboratory data in the studied groups

Parameters	Patient	Control	*P* value
Age (years)	3.8 ± 2.7	4 ± 2.9	0.514
Sex (male:female)	15:25	17:23	0.648
Weight (kg)	8.1 ± 2.5	9.3 ± 2.2	0.028
HR (beat/min)	116.7 ± 17.9	101.9 ± 9.4	< 0.001
RR (cycle/min)	40.9 ± 10.2	34.6 ± 6.7	0.001
LV FS	19.6 ± 4.2	39.3 ± 4.2	< 0.001
Mitral *E/A* ratio	1.1 ± 0.2	1.5 ± 0.1	0.001
Diagnosis			
Cardiomyopathy	13 (32.5%)		
CHD	27 (67.5%)		
ROSS classification			
Class I	0 (0%)	40 (100%)	
Class II	7 (17.5%)	0 (0%)	
Class III	16 (40%)	0 (0%)	
Class IV	17 (42.5%)	0 (0%)	
Copeptin levels (pmol/L)	16.2 ± 5	4.1 ± 2.3	< 0.001

*HR* heart rate, *RR* respiratory rate, *LV FS* left ventricular fraction shortening, *E/A ratio* E wave is a peak early filling velocity and A wave is a peak late filling velocity, *CHD* congenital heart disease

Copeptin level was significantly higher in patients with Ross class IV (19.6 ± 3.9) compared to those with class III (15.2 ± 4) and class II (10.4 ± 1.5), *P* ˂ 0.001 (Table 2).

**Table 2:** Copeptin levels in different Ross classification in the patient group

Parameter	Class II (*n* = 7)	Class III (*n* = 16)	Class IV (*n* = 17)	*P* value
Copeptin level (pmol/L)	10.4 ± 1.5	15.2 ± 4	19.6 ± 3.9	< 0.001
Post Hoc test	II vs III	II vs IV	III vs IV	
	*P*1 = 0.018	*P*2 ≤ 0.001	*P*3 = 0.004	

The mortality rate in our study was 7.5% (three patients); readmission within the period of follow-up occurred in another nine patients (22.5%). Moreover, copeptin levels were significantly higher in patients with bad prognosis (21.2 ± 4.1) compared to those with good prognosis (14.5 ± 4.1), *P* ˂ 0.001. (Table 3).

**Table 3:** Copeptin levels in patients with good and bad prognosis in the patient group

Patients with HF (*n* = 40)	Number	Percentage (%)	Copeptin level
Patients with bad prognosis	12	30	21.2±4.1
Death	3	7.5	
Readmission	9	22.5	
Patients with good prognosis	28	70	14.5 ± 4.1
*P* value	< 0.001		

*HF* heart failure

Copeptin level had a significant positive correlation with age, HR, RR, and ROSS classification. On the contrary, copeptin level had a significant negative correlation with LV FS and mitral E/A ratio. Moreover, copeptin level had no significant correlation with sex, weight, or diagnosis. (Table 4).

**Table 4:** Correlation between copeptin levels and various clinical and echocardiographic data

Parameters	Copeptin (pmol/L)	
	*R*	*P*
Age	0.247	0.027
Sex	0.089	0.431
HR	0.460	< 0.001
RR	0.404	< 0.001
Weight	−0.050	0.661
LV FS	−0.826	< 0.001
Mitral *E/A* ratio	−0.811	< 0.001
Diagnosis	0.153	0.631
Ross classification	0.897	˂ 0.001

*HR* heart rate, *RR* respiratory rate, *LV FS* left ventricular fraction shortening, *E/A ratio* E wave is a peak early filling velocity and A wave is a peak late filling velocity

**Fig. 1:**
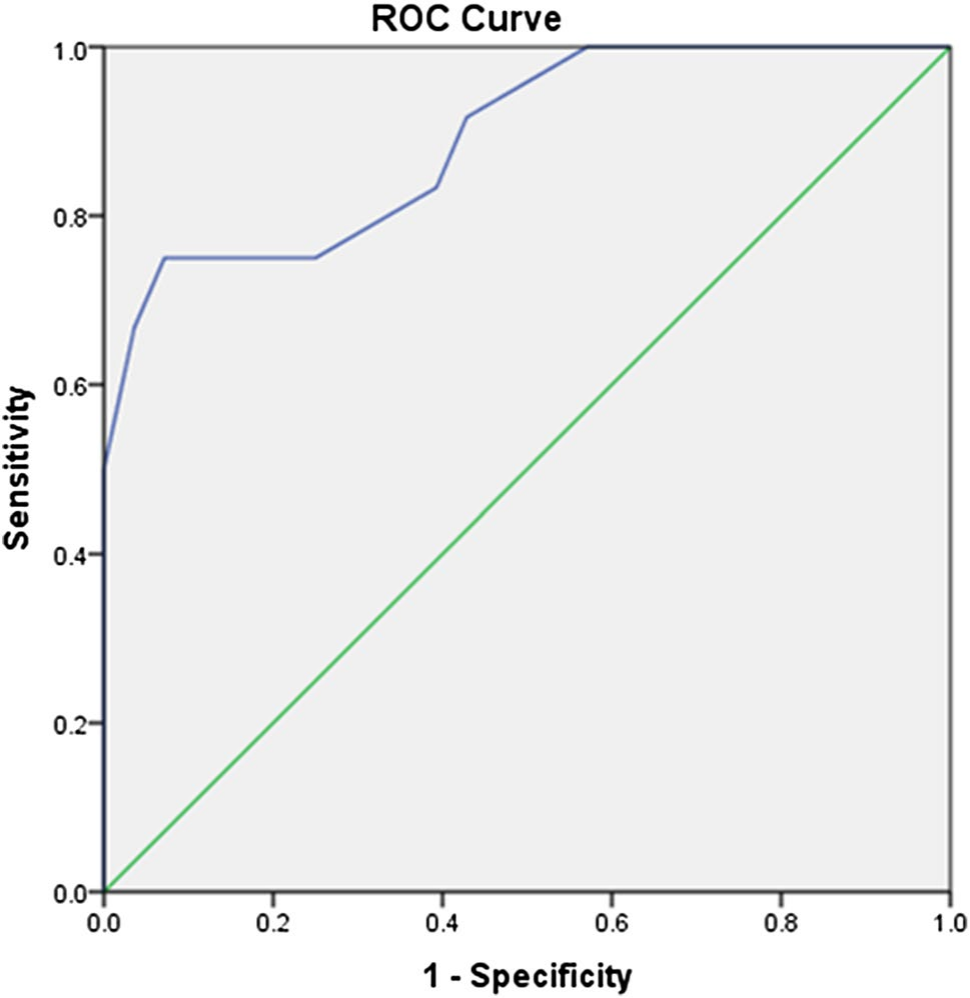
ROC curve to assess the predictive value of copeptin level to predict adverse outcome in children with acute HF

ROC curve showed that the cut-off value of ≥ 19.5 pmol/L yielded a sensitivity of 75% and a specificity of 93% to predict adverse outcome in children with acute HF. (Fig. 1).

## Discussion

The Predictive role of plasma copeptin levels has been studied in several studies in adults. However, its value as a predictive biomarker for HF is still under evaluated in pediatrics.

In our study, we found that the mean copeptin levels were significantly higher in children with HF compared to the control group indicating that copeptin level can be used as a marker for the diagnosis of HF. Similar result was documented in adults with HF [14, 15]. Recently, a preprint study in pediatrics showed that copeptin levels were elevated in children with HF compared to healthy control [16]. Moreover, Karki et al. [17] found that copeptin levels were higher in pediatric patients with HF due to cardiomyopathy compared to the control group. During HF, low cardiac output and atrial pressure stimulate neurohormonal reflexes with a subsequent increase of VP secretion that explain the high level of copeptin in HF [18].

Copeptin level was found to positively correlate with the severity of HF presented by ROSS classification being the highest in patients with class IV and the lowest in patients with class II which reflected the role of copeptin in pathogenesis and identification of the severity of HF. Similar results were reported by other researchers [14, 17]. Increased copeptin level could be used as a marker of more advanced HF and inevitable hospitalization [19].

Moreover, copeptin level was inversely correlated with LV FS and mitral *E/A* ratio indicating that copeptin level was higher with more advanced HF. Whether it is a cause or effect, this needs more research to investigate this point. In addition to the well-known vasoconstriction and water retention effects of vasopressin, it has been proposed to exert direct effects on the myocardium leading to left ventricular hypertrophy and remodeling with a subsequent negative effects on myocardial contractility [20, 21].

Copeptin level was also found to be positively correlated with age, but whether this was due to the effect of more advanced disease or the effect of the age per se is still unclear. Future studies are required to address this question.

Interestingly, copeptin levels were significantly higher in children with poor prognosis (mortality and readmission) compared to those with good prognosis. Moreover, copeptin at a cut-off value of ≥ 19.5 pmol/L yielded a sensitivity of 75% and a specificity of 93% to predict poor prognosis in children with HF. In line with our results, copeptin was found to be a strong predictor for all case mortality/hospitalization by earlier studies in adults [22–29]. Copeptin serves as a surrogate biomarker for VP levels. Accordingly, elevated plasma levels of copeptin reflect an unfavorable hemodynamic profile and a poor outcome in patients with HF [16, 17, 28].

HF is characterized by a high rate of mortality and morbidity in children thus finding an easy dependable marker for identifying high risk patients who need early intervention and more intense treatment is crucial to improve outcome and to decrease mortality.

Limitation of the study: relatively small number of patients, being a single center study, serial measurement of copeptin level was not performed, and diagnostic value of copeptin level to predict the response of treatment in such patients was not evaluated.

## Conclusion

Plasma copeptin level has a good prognostic value to predict adverse outcome in pediatric HF. Moreover, copeptin correlated well with the severity of pediatric HF.

## Data Availability

Available when requested.
